# The C-reactive protein/albumin ratio, a validated prognostic score, predicts outcome of surgical renal cell carcinoma patients

**DOI:** 10.1186/s12885-017-3119-6

**Published:** 2017-03-06

**Authors:** Shengjie Guo, Xiaobo He, Qian Chen, Guangwei Yang, Kai Yao, Pei Dong, Yunlin Ye, Dong Chen, Zhiling Zhang, Zike Qin, Zhuowei Liu, Yunfei Xue, Meng Zhang, Ruiwu Liu, Fangjian Zhou, Hui Han

**Affiliations:** 1 Department of Urology, Sun Yat-Sen University Cancer Center, State Key Laboratory of Oncology in South China, Collaborative Innovation Center for Cancer Medicine, 651 Dongfeng Road East, Guangzhou, Guangdong 510060 People’s Republic of China; 2 Department of Medical Oncology, the Fifth Affiliated Hospital of Sun Yat-Sen University, Zhuhai, China; 3 Xinhua college of Sun Yat-Sen University, Guangzhou, China; 4 Medicine school of Sun Yat-Sen University, Guangzhou, China; 5 Department of Biochemistry and Molecular Medicine, University of California Davis, Sacramento, CA USA

**Keywords:** C-reactive protein/albumin ratio, Prognostic score, Renal cell carcinoma, Surgical resection

## Abstract

**Background:**

The preoperative C-reactive protein/Albumin (CRP/Alb) ratio has been shown to be valuable in predicting the prognosis of patients with certain cancers. The aim of our study is to explore its prognostic value in patients with renal cell carcinoma (RCC).

**Methods:**

A retrospective study was performed in 570 RCC patients underwent radical or partial nephrectomy including 541 patients who received full resection of localized (T1-3 N0/+ M0) RCC. The optimal cutoff value of CRP/Alb was determined by the receive operating characteristic (ROC) analysis. The impact of the CRP/Alb and other clinicopathological characteristics on overall survival (OS) and disease-free survival (DFS) was evaluated using the univariate and multivariate Cox regression analysis.

**Results:**

The optimal cutoff of CRP/Alb ratio was set at 0.08 according to the ROC analysis. Multivariate analysis indicated that CRP/Alb ratio was independently associated with OS of RCC patients underwent radical or partial nephrectomy (hazard ratio [HR]: 1.94; 95% confidence interval [95% CI]: 1.12–3.36; *P* = 0.018), and DFS of localized RCC patients underwent full resection (HR: 2.14; 95% CI: 1.22–3.75; *P* = 0.008).

**Conclusion:**

Elevated CRP/Alb ratio was an independent prognostic indicator for poor OS in patients underwent radical or partial nephrectomy and DFS of localized RCC patients underwent full resection. Overall, CRP/Alb may help to identify patients with high relapse risk.

**Electronic supplementary material:**

The online version of this article (doi:10.1186/s12885-017-3119-6) contains supplementary material, which is available to authorized users.

## Background

Renal cell carcinoma (RCC) is the most common malignancy in females with urological tumors and ranks the third place in males after prostate and bladder cancers [1]. Broad applications of radiological technologies especially abdominal ultrasound or computerized tomography have led to increase in detection of renal tumors in relatively small size and localized in the kidney [2]. Patients with localized diseases usually undergo curative whole or partial nephrectomy. However, up to 40% patients will eventually relapse with secondary tumors at distant sites [3, 4]. At first presentation, one-third of all RCC patients will have established metastatic renal cell carcinoma (mRCC). Despite the introduction of molecular targeted therapies, the overall 5-year survival rate of this patient group rarely exceeds 10% [5, 6]. In addition, RCC is characterized by chemo- and radio-resistance. The clinical course in localized RCC is difficult to predict, even within patients who have similar clinic-pathological parameters, such as tumor stage and grade [7, 8]. Therefore, it is important to identify promising prognostic factors to guide patient management after curative surgery treatment.

Increasing evidences have demonstrated the role of inflammation in carcinogenesis and tumor progression. The prognostic value of many inflammation-based scores, such as preoperative C-reactive protein (CRP), the Glasgow Prognostic Score (GPS), modified Glasgow prognostic score (mGPS), high-sensitivity modified Glasgow prognostic score (HS-mGPS), neutrophil to lymphocyte ratio (NLR), platelet to lymphocyte ratio (PLR), and systemic immune-inflammation index (SII), has been validated in many types of cancer, including RCC [9–13]. Additionally, some studies have demonstrated that the preoperative nutritional status, such as hypoalbuminemia, weight loss and low body mass index (BMI), are associated with worse outcomes of RCC patients after radical or partial nephrectomy [14, 15]. Recently, a new prognostic index, preoperative C-reactive protein/albumin (CRP/Alb) ratio, in combination with the systemic inflammation and nutritional status, has also been reported as an independent prognostic marker in hepatocellular cancer (HCC), gastric cancer (GC) and small-cell lung cancer (SCLC) [16–18]. Although Chen et al. reported the prognostic influence of CRP/Alb ratio on overall survival (OS) of patients with clear cell renal carcinoma [19], its prognostic role in RCC still need to be further explored. In this retrospective study, we examined the prognostic value of CRP/Alb ratio in patients with RCC and investigated the relationship between CRP/Alb ratio and the clinical outcomes of RCC patients.

## Methods

### Patients

We executed a retrospective cohort study of 912 consecutive RCC patients who underwent radical or partial nephrectomy between January 2000 and December 2012 in Sun Yat-sen University Cancer Center (SYSUCC). The inclusion criteria were as follows: 1) patients were cytologically or histologically diagnosed with RCC; 2) data on complete blood laboratory measurements included serum CRP and albumin (Alb) within one week before performing radical or partial nephrectomy. Patients without blood laboratory measurements prior to surgical resection, patients with active inflammatory disease and patients with other malignancies were excluded from the study. At last, a total of 570 patients were enrolled in the study. This retrospective study was conducted in accordance with the standards of the Declaration of Helsinki and was approved by the Sun Yat-sen University Cancer Center research ethics committee (Number: GZR2016-100). All patients have provided written informed consent for their information to be stored and used in the hospital database.

### Clinical data extraction

The baseline clinical and pathologic characteristics were collected, including age at the time of surgery, gender, BMI, lactate dehydrogenase (LDH), urine protein, alkaline phosphatase (ALP), serum creatinine (CRE), uric acid (UA), total protein, serum globulin, neutrophil count, lymphocyte count, platelet count, disease stage and histology by using a standard data extraction system. Elevated ALP level was defined as serum ALP > 135 U/L. Elevated LDH was defined as serum LDH > 245 U/L. Elevated CRE was defined as serum CRE > 130 μmol/L. Elevated UA was defined as UA > 420 μmol/L. Elevated total protein was as total protein > 80 g/L. Elevated globulin was defined as globulin > 35 g/L. Tumor stage was determined based on the 2010 TNM classification of malignant tumors staging system and tumor grade was defined according to the Fuhrman grading system. All the blood samples were tested prior to initial treatment. The NLR, PLR and CRP/Alb ratio were calculated based on the following equations, respectively.

$$ \mathrm{N}\mathrm{L}\mathrm{R} = \kern0.5em \mathrm{neutrophil}\ \mathrm{count}\ \mathrm{t}\mathrm{o}\ \mathrm{lymphocyte}\ \mathrm{count}, $$

$$ \mathrm{P}\mathrm{L}\mathrm{R} = \mathrm{platelet}\ \mathrm{count}\ \mathrm{t}\mathrm{o}\ \mathrm{lymphocyte}\ \mathrm{count}, $$

$$ \mathrm{C}\mathrm{R}\mathrm{P}/\mathrm{Alb}=\mathrm{t}\mathrm{he}\ \mathrm{serum}\ \mathrm{C}\mathrm{R}\mathrm{P}\ \mathrm{level}\ \mathrm{t}\mathrm{o}\ \mathrm{t}\mathrm{he}\ \mathrm{serum}\ \mathrm{Alb}\ \mathrm{level}. $$

### Patients follow-up

A dynamic computed tomogram was performed every 3 months in two years, 6 months in 2–5 years and 1 year after 5 years. The last survival follow-up date was November 01, 2015. Overall survival (OS) was calculated from the date of surgery to the date of death or last follow-up. Disease-free survival (DFS) was calculated from the date of surgery to the date of disease recurrence or metastasis or the last follow-up in localized RCC patients who underwent full resection.

### Statistical analysis

Descriptive statistics of patients’ characteristics (i.e. age and BMI) were presented as mean ± SD (standard deviation). Comparisons between groups were performed using the Kruskal-Wallis or *χ*2 test. Pearson correlation was performed to evaluate the relationship of serum CRP and Alb with OS. The optimal cut-off points for the inflammation-based factors were determined by receive operating characteristic (ROC) analysis and the areas under the curve (AUC) were calculated. Survival analysis and curves were performed according to the Kaplan-Meier method and compared by the log-rank test. A Cox proportional-hazard model for multivariable analysis was applied for variables that proved to be significant in the univariate analysis. Hazard ratios (HR) with 95% confidence interval (95% CI) were also calculated using univariate or multivariate analysis. If variables were significantly associated with other variables, they were excluded from the final multivariable analysis. Statistical analyses were performed using IBM SPSS 21.0 software (IBM Corporation, Armonk, NY). Differences at *p* < 0.05 were considered to be significant in all statistical analyses.

## Results

### Patient demographics and outcomes

The clinicpathological characteristics of the 570 RCC patients were summarized in Table 1. Their mean age was 51.43 ± 13.52 years old and their mean and median follow-up periods were 65.19 and 63.54 months, respectively. Among them, 382 (67%) were males and 188 (33%) were females; 81 (14.2%) died and 489 (85.8%) survived at last follow-up. There were 451 (79.10%) patients with clear cell, 41 (7.20%) with papillary, 78 (13.70%) with others RCC (such as 27 (4.70%) with chromophobe, 10 (1.80%) with multilocular cystic, 41 (7.20%) with other histological types of RCC). The overall cancer-specific survival (CSS) was 93.1% at 1 year, 89.6% at 2 years, and 81.6% at 5 years.

**Table 1:** Baseline characteristics of all patients (*n* = 570)

Characteristics	Cases (*n* = 570)	Percentage (%)
Age (years) (Mean ± SD)	51.43 ± 13.52	
BMI (Mean ± SD)	23.57 ± 3.59	
Gender		
Male	382	67.00
Female	188	33.00
Pathological types		
Clear cell carcinoma	451	79.10
Papillary carcinoma	41	7.20
Others	78	13.70
Fuhrman-grade		
I	119	20.90
II	249	43.70
III	60	10.50
IV	7	1.20
Unknown	135	23.70
pTNM stage		
I	397	69.60
II	85	14.90
III	59	10.40
IV	29	5.10
pT status		
T1	407	71.40
T2	94	16.50
T3+ T4	69	12.10
pN status		
N0	535	93.90
N1	35	6.10
pM status		
M0	550	96.50
M1	20	3.50
Urine protein		
No	460	80.70
Yes	28	4.90
Unknown	82	14.40
ALP		
Normal	525	92.10
Elevated	45	7.90
LDH		
Normal	468	82.10
Elevated	102	17.90
CRE		
Normal	546	95.80
Elevated	24	4.20
UA		
Normal	461	80.90
Elevated	109	19.10
Total protein		
Normal	503	88.20
Elevated	67	11.80
Serum globulin		
Normal	360	63.20
Elevated	210	36.80
NLR		
< 1.85	242	42.50
≥ 1.85	328	57.50
PLR		
< 153	418	73.30
≥ 153	152	26.70
CRP/Alb		
< 0.08	393	68.90
≥ 0.08	177	31.10

*Abbreviation*: *BMI* body mass index, *pTNM* pathologic tumor–node–metastasis, *ALP* alkaline phosphatase, *LDH* lactate dehydrogenase, *CRE* serum creatinine, *UA* uric acid, *LDH* lactate dehydrogenase, *NLR* neutrophil count to lymphocyte count, *PLR* platelet count to lymphocyte count, *CRP/Alb* the serum CRP level to the serum Alb level

### The relationship of serum CRP and Alb with OS

We explored the association of the serum CRP and Alb with OS. The results showed a significant negative correlation between serum CRP level and OS (r = −0.141, *P* < 0.001) (Additional file 1: Figure S1a) and a significant positive correlation between serum Alb level and OS (r =0.317, *P* < 0.001) (Additional file 1: Figure S1b).

### The optimal cut-off value of inflammation-based factors by the ROC analysis

**Fig. 1:**
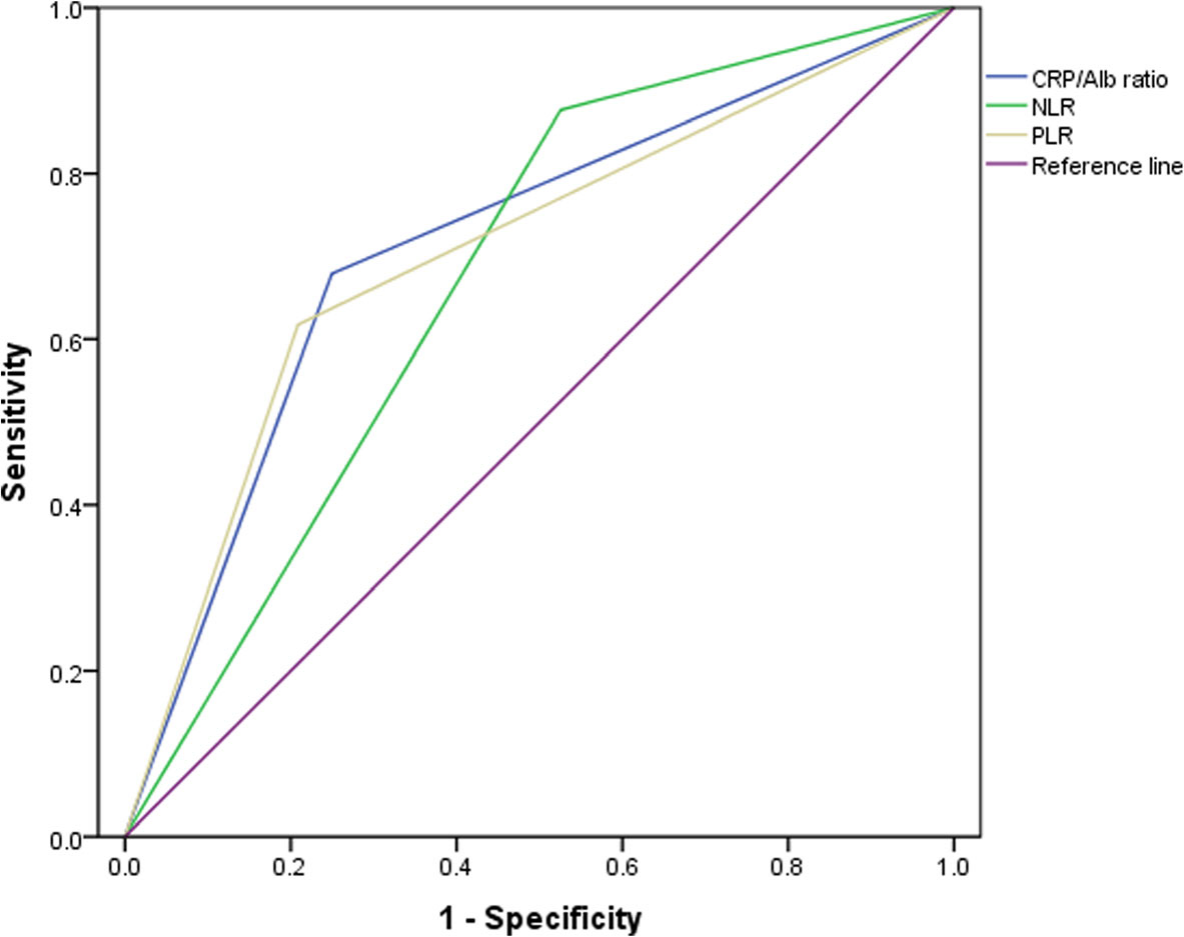
The predictive ability of the preoperative NLR, PLR and CRP/Alb ratio was compared by ROC curves

Based on the area under ROC curve (AUC) of 0.715 (*P* < 0.001) for survival in the ROC analysis, the optimal cut-off value was 0.08 for CRP/Alb ratio. 1.85 for NLR and 153 for PLR, respectively. The sensitivity and specificity of CRP/Alb ratio were 66.7 and 75.1%, respectively. In addition, the ability to distinguish CRP/Alb ratio from other inflammation-based prognostic factors was compared using the levels of AUC. The results showed that was 0.675 and 0.704 for NLR and PLR, respectively (Fig. 1 and Additional file 2: Table S1). Based on the cut-off value of CRP/Alb ratio, 177(31.1%) patients were assigned into low CRP/Alb group and 393 (68.9%) patients were in the high CRP/Alb group.

### The clinicopathological characteristics and the preoperative CRP/Alb ratio

The clinicopathological characteristics of all patients are described in Table 2. An elevated CRP/Alb ratio was significantly associated with the Fuhrman-grade (*P* < 0.001), T stage (*P* < 0.001), N stage (*P* < 0.001), M stage (*P* < 0.001), ALP (*P* = 0.018), LDH (*P* = 0.004), NLR (*P* < 0.001) and PLR (*P* < 0.001). For patients in the low CRP/Alb ratio group, 94.7% were at stag T1/T2 and 5.3% at stage T3/T4. However, for patients in the high CRP/Alb ratio group, only 72.9% patients were at stage T1/T2 and 27.1% at T3/T4 (*P* < 0.001). Similarly, the percentage of patients at stage N0/N1 was 97.2%/2.8% and that of patients at stage M0/M1 was 99.2%/0.8% in patients in the low CRP/Alb ratio group. By comparison, the percentage of patients at N0/N1 was 86.4%/13.6% and at stage M0/M1 was 90.4%/9.6% in the high CRP/Alb ratio group (*P* < 0.001). These results indicate that CRP/Alb ratio is associated with the disease progression and low CRP/Alb ratio is related with the early stage of RCC.

**Table 2:** Clinicopathological variables of patients according to the cutoff value of CRP/Alb ratio

Characteristics	CRP/Alb < 0.08 (*n* = 393)	CRP/Alb ≥ 0.08 (*n* = 177)	*P* value
Age (years)	48.71 ± 12.98	55.25 ± 13.92	<0.001^a^
BMI	23.65 ± 3.37	23.41 ± 4.02	0.290^a^
Gender			0.219^b^
Male	257 (65.40%)	125 (70.60%)	
Female	136 (34.60%)	52 (29.40%)	
Pathological types			0.315^b^
Clear cell carcinoma	308 (81.20%)	43 (80.80%)	
Papillary carcinoma	26 (3.80%)	15 (8.50%)	
Others	59 (15.00%)	19 (10.70%)	
Fuhrman-grade			<0.001^b^
I	92 (23.40%)	27(15.30%)	
II	178(45.30%)	71(40.10%)	
III	31(7.90%)	29(16.40%)	
IV	2(0.50%)	5(2.80%)	
Unknown	90(22.90%)	45(25.40%)	
pTNM stage			<0.001^b^
I	313(79.60%)	84(47.50%)	
II	53(13.50%)	32(18.10%)	
III	22(5.60%)	37(20.90%)	
IV	5 (1.30%)	24(13.60%)	
pT status			<0.001^b^
T1	318 (80.90%)	89 (50.30%)	
T2	54 (13.70%)	40 (22.60%)	
T3+ T4	21 (5.30%)	48 (27.10%)	
pN status			<0.001^b^
N0	382 (97.20%)	153 (86.40%)	
N1	11 (2.80%)	24 (13.60%)	
pM status			<0.001^b^
M0	390 (99.20%)	160 (90.40%)	
M1	3 (0.80%)	17 (9.60%)	
Urine protein			0.188^b^
No	319 (81.20%)	141 (79.7%)	
Yes	15 (3.80%)	13 (7.3%)	
Unknown	59 (15.00%)	23 (13.00%)	
ALP			0.018^b^
Normal	369 (93.90%)	156 (88.10%)	
Elevated	24 (6.10%)	21 (11.90%)	
LDH			0.004^b^
Normal	335 (85.20%)	133 (75.10%)	
Elevated	58 (14.80%)	44 (24.90%)	
CRE			0.001^b^
Normal	384 (97.70%)	162 (91.50%)	
Elevated	9 (2.30%)	15 (8.50%)	
UA			0.468^b^
Normal	321 (81.70%)	140 (79.10%)	
Elevated	72 (18.30%)	37 (20.90%)	
Total protein			0.144^b^
Normal	352 (89.60%)	151 (85.30%)	
Elevated	41 (10.40%)	26 (14.70%)	
Serum globulin			
Normal	273 (69.50%)	87 (49.20%)	<0.001^b^
Elevated	120 (30.50%)	90 (50.80%)	
NLR			<0.001^b^
< 1.85	205 (52.20%)	37 (20.90%)	
≥ 1.85	188 (47.80%)	140 (79.10%)	
PLR			<0.001^b^
< 153	324 (82.40%)	94 (53.10%)	
≥ 153	69 (17.60%)	83 (46.90%)	

*Abbreviation*: *BMI* body mass index, *pTNM* pathologic tumor–node–metastasis, *ALP* alkaline phosphatase, *LDH* lactate dehydrogenase, *CRE* serum creatinine, *UA* uric acid, *LDH* lactate dehydrogenase, *NLR* neutrophil count to lymphocyte count, *PLR* platelet count to lymphocyte count, *CRP/Alb* the serum CRP level to the serum Alb level

^a^Kruskal-Wallis test

^b^Chi-square test

### The relationship between the preoperative CRP/Alb ratio and OS in all RCC patients

**Fig. 2:**
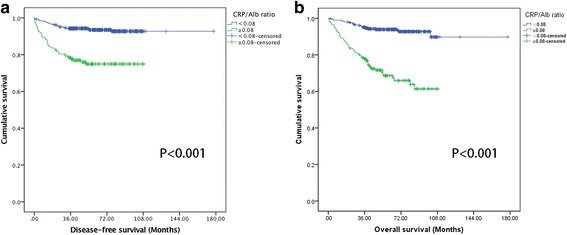
Kaplan-Meier curves depicting DFS (*n* = 541) and OS (*n* = 570) according to the preoperative optimal value of CRP/Alb in patients with renal cell carcinoma. **a** Kaplan-Meier analysis of DFS in 541 patients. **b** Kaplan-Meier analysis of OS in 570 patients

**Fig. 3:**
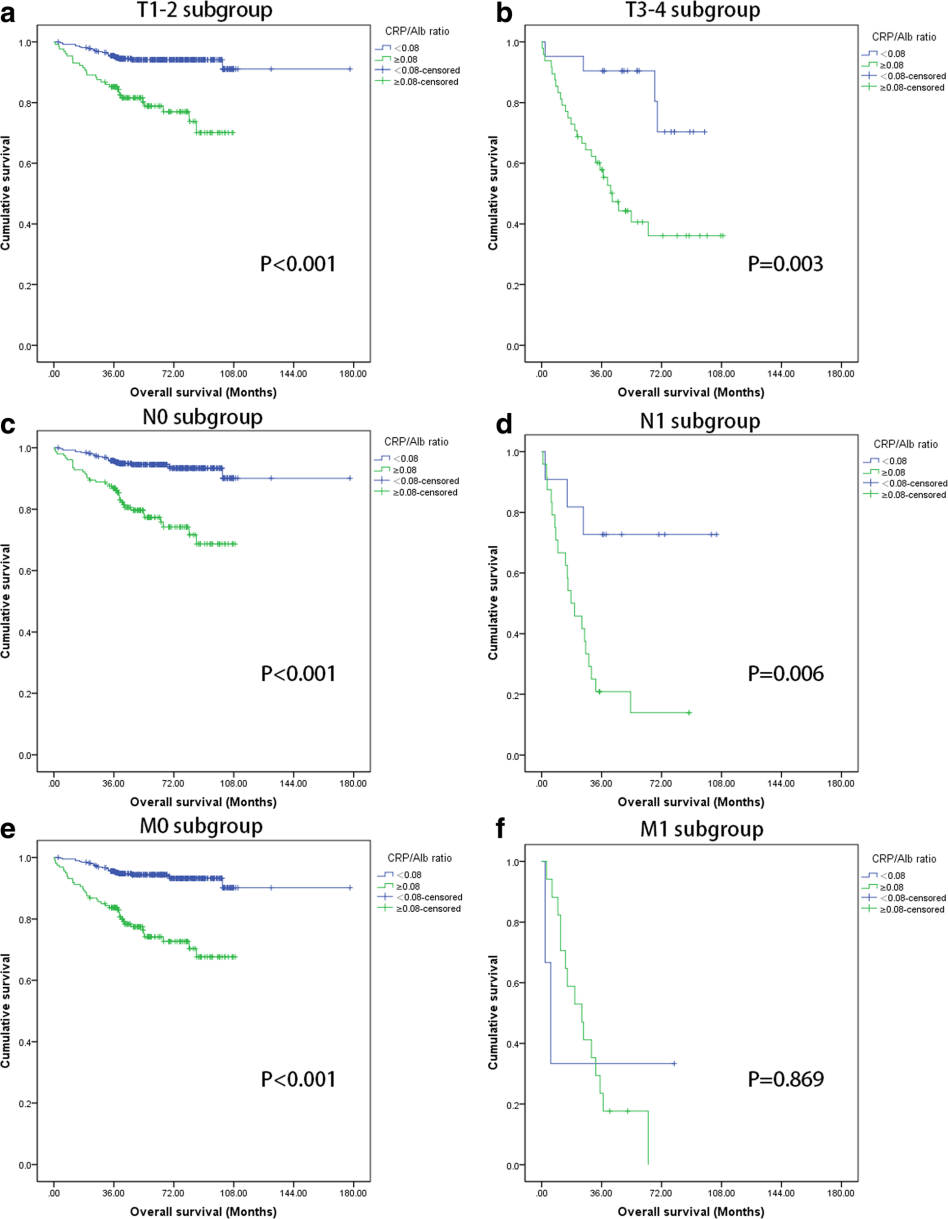
Kaplan-Meier curves showing OS according to the preoperative optimal value of CRP/Alb in 570 patients with renal cell carcinoma. Patients were stratified according to the pT-status, pN-status, and pM-status. **a** Kaplan-Meier analysis of OS in T1-2 subgroup. **b** Kaplan-Meier analysis of OS in T3-4 subgroup. **c** Kaplan-Meier analysis of OS in N0 subgroup. **d** Kaplan-Meier analysis of OS in N1 subgroup. **e** Kaplan-Meier analysis of OS inM0 subgroup. **f** Kaplan-Meier analysis of OS in M1 subgroup

Compared with high CRP/Alb ratio, patients with low CRP/Alb ratio had longer OS (CRP/Alb˂0.08 vs. ≥0.08, mean OS: 164.87 vs 79.92 months, P˂0.001) (Fig. 2b). Similarly, longer OS was also observed in patients in the low CRP/Alb group at early stage T1/T2 (P˂0.001), at the advanced stage T3/T4 (*P* = 0.003), at N0 (P˂0.001), N1(*P* = 0.006), M0 (P˂0.001) stages, but not at M1stage (*P* = 0.869) (Fig. 3).

Table 3 shows the results of the univariate and multivariate analysis of OS. It is clear from the univariate analysis that the CRP/Alb ratio is associated with the OS of RCC patients (HR: 5.55; 95% CI: 3.48–8.86; *P* < 0.001). After excluding the related variables, the significant variables (age, BMI, T stage, N stage, M stage, NLR, PLR and CRP/Alb ratio) were tested in the multivariate analysis. The multivariate analysis indicated that the CRP/Alb ratio (HR: 1.94; 95% CI: 1.12–3.36; P =0. 018) is an independent prognostic factor for OS in addition to N stage (HR: 3.62; 95% CI: 1.91–6.85; *P* < 0.001), M stage (HR: 3.12; 95% CI: 1.57–6.19; *P* = 0.001) and PLR (HR: 2.42; 95% CI: 1.43–4.07; *P* < 0.001), but not LDH and NLR.

**Table 3:** Univariate and multivariate analyses for variables considered for overall survival (OS) (Cox proportional hazard regression model) (*n* = 570)

	OS Univariate analysis			OS Multivariate analysis		
Characteristics	95% CIs	HR	*P* value	95% CIs	HR	*P* value
Age (years)	1.01 to 1.05	1.03	<0.001^a^	1.01 to 1.05	1.03	<0.001^b^
BMI	0.82 to 0.93	0.87	<0.001^a^	0.84 to 0.97	0.91	0.007^b^
Gender						
Male		1.00(ref.)				
Female	0.73 to 1.82	1.16	0.532^a^			
Pathological types						
Clear cell carcinoma		1.00(ref.)			1.00(ref.)	
Papillary carcinoma	1.36 to 4.72	2.53	0.003^a^	0.67 to 4.39	1.72	0.258^b^
Others	0.70 to 2.42	1.30	0.414^a^	0.64 to 3.99	1.60	0.312^b^
Fuhrman-grade						
I		1.00(ref.)			1.00(ref.)	
II	0.68 to 2.82	1.39	0.365^a^	0.72 to 3.21	1.52	0.269^b^
III	1.46 to 7.14	3.23	0.004^a^	0.57 to 3.09	1.33	0.507^b^
IV	1.70 to 22.12	6.14	0.006^a^	0.43 to 6.08	1.62	0.474^b^
unknown	1.25 to 5.19	2.55	0.010^a^	0.43 to 2.55	1.05	0.922^b^
pTNM stage						
I		1.00(ref.)				
II	1.27 to 4.85	2.48	0.008^a^			
III	3.51 to 11.40	6.33	<0.001^a^			
IV	14.54 to 46.54	26.01	<0.001^a^			
pT status						
T1		1.00(ref.)			1.00(ref.)	
T2	1.76 to 5.46	3.10	<0.001^a^	1.20 to 3.90	2.16	0.011^b^
T3+ T4	4.72 to 12.92	7.81	<0.001^a^	0.91 to 3.13	1.69	0.098^b^
pN status						
N0		1.00(ref.)			1.00(ref.)	
N1	6.42 to 17.09	10.47	<0.001^a^	1.91 to 6.85	3.62	<0.001^b^
pM status						
M0		1.00(ref.)			1.00(ref.)	
M1	8.71 to 26.12	15.08	<0.001^a^	1.57 to 6.19	3.12	0.001^b^
Urine protein						
No		1.00(ref.)				
Yes	0.46 to 2.84	1.14	0.773^a^			
Unknown	0.48 to 1.74	0.92	0.787^a^			
ALP						
Normal		1.00(ref.)				
Elevated	0.61 to 2.63	1.27	0.527^a^			
LDH						
Normal		1.00(ref.)				
Elevated	0.84 to 2.34	1.40	0.203^a^			
CRE						
Normal		1.00(ref.)				
Elevated	0.78 to 4.07	1.78	0.175^a^			
UA						
Normal		1.00(ref.)				
Elevated	0.77 to 4.08	1.78	0.176^a^			
Total protein						
Normal		1.00(ref.)				
Elevated	0.77 to 2.54	1.40	0.265^a^			
Serum globulin						
Normal		1.00(ref.)			1.00(ref.)	
Elevated	1.66 to 4.03	2.59	<0.001^a^	0.84 to2.31	1.39	0.203^b^
NLR						
< 1.85		1.00(ref.)			1.00(ref.)	
≥ 1.85	3.08 to 11.59	5.97	<0.001^a^	0.97 to 4.32	2.05	0.060^b^
PLR						
< 153		1.00(ref.)			1.00(ref.)	
≥ 153	3.49 to 8.56	5.46	<0.001^a^	1.43 to 4.07	2.42	<0.001^b^
CRP/Alb						
< 0.08		1.00(ref.)			1.00(ref.)	
≥ 0.08	3.48 to 8.86	5.55	<0.001^a^	1.12 to 3.36	1.94	0.018^b^

*Abbreviation*: *HR* hazard ratio, *CIs* confidence intervals, *BMI* body mass index, *pTNM* pathologic tumor–node–metastasis, *ALP* alkaline phosphatase, *LDH* lactate dehydrogenase, *CRE*, serum creatinine, *UA*, uric acid, *LDH* lactate dehydrogenase, *NLR* neutrophil count to lymphocyte count, *PLR* platelet count to lymphocyte count, *CRP/Alb* the serum CRP level to the serum Alb level

^a^Univariate Cox proportional hazard regression

^b^Multivariate Cox proportional hazard regression

### The relationship between the preoperative CRP/Alb ratio and DFS in localized (T1-3 N0/+ M0) RCC patients underwent full resection

**Fig. 4:**
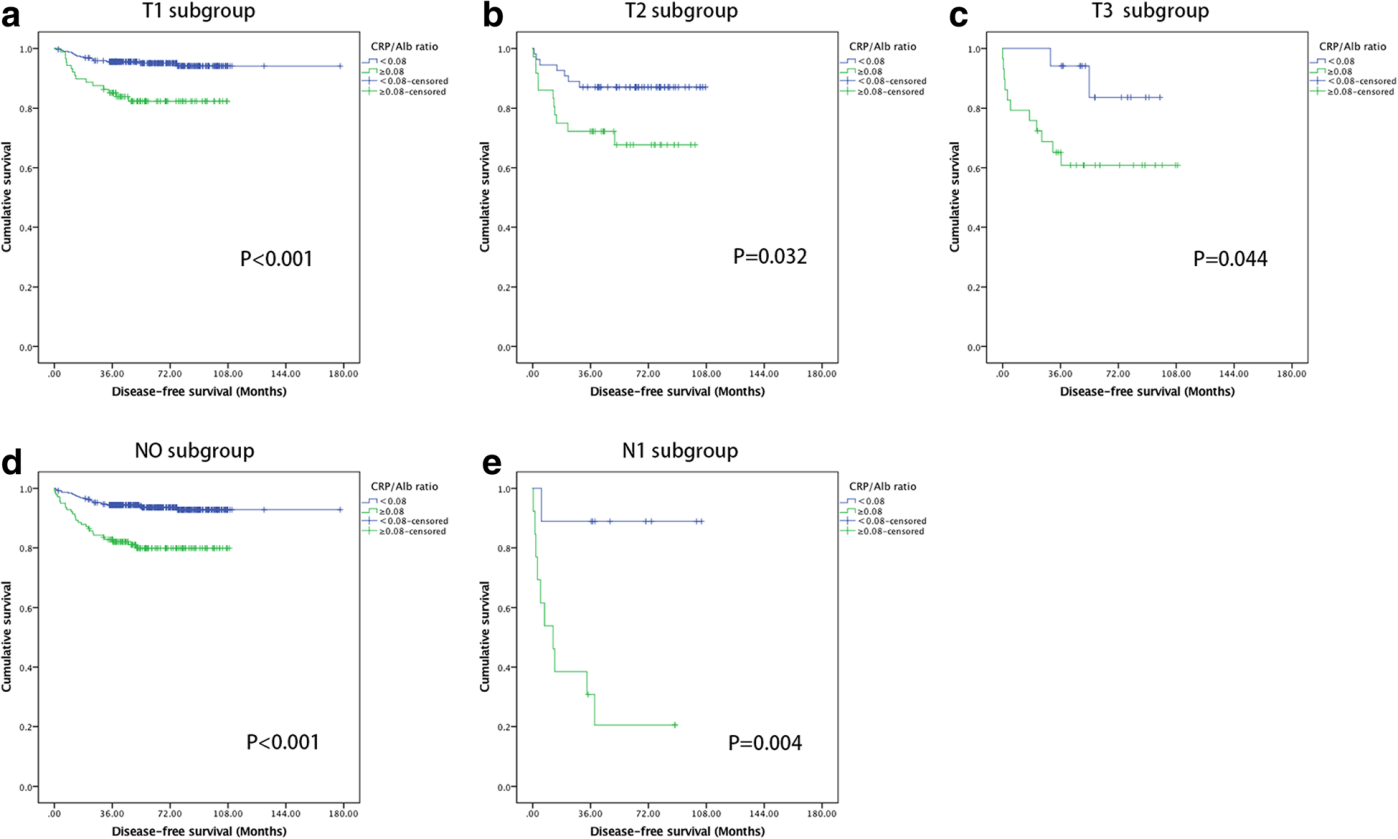
Kaplan-Meier curves showing DFS according to the preoperative optimal value of CRP/Alb in 541 patients with renal cell carcinoma. Patients were stratified according to the pT-status, pN-status. **a** Kaplan-Meier analysis of DFS in T1 subgroup. **b** Kaplan-Meier analysis of DFS in T2 subgroup. **c** Kaplan-Meier analysis of DFS in T3 subgroup. **d** Kaplan-Meier analysis of DFS in N0 subgroup. **e** Kaplan-Meier analysis of DFS in N1 subgroup

The clinicopathological characteristics of 541 localized (T1-3 N0/+ M0) RCC patients underwent full resection were summarized in Additional file 3: Table S2. CRP/Alb ratio was used to analyze the DFS of these patients, who were considered as received the curative treatment. Among them, patients with low CRP/Alb ratio had longer DFS event than patients in the high CRP/Alb ratio group (CRP/Alb˂0.08 vs. ≥0.08, mean DFS: 166.75 vs 85.58 months, P˂0.001) (Fig. 2a). In addition, DFS of patients at stages T1, T2, T3,N0 and N1 in the low CRP/Alb ratio group also had longer DFS event than patients in the high CRP/Alb ratio group (*P* < 0.001, *P* = 0.032, *P* = 0.044, *P* < 0.001 and *P* = 0.004, respectively) (Fig. 4).

Table 4 showed the results of univariate and multivariate analyses of DFS. It is clear from the univariate analysis results that CRP/Alb ratio is associated with DFS of localized RCC patients underwent full resection (HR: 4.22; 95% CI: 2.54–7.02; P <0.001). The multivariate analysis also indicated that CRP/Alb ratio (HR: 2.14; 95% CI: 1.22–3.75; P = 0.008) is an independent prognostic factor for DFS of these patients. In addition, age (HR: 1.03; 95% CI: 1.01–1.06; P <0.001), BMI (HR: 0.89; 95% CI: 0.82–0.97; P = 0.005), N stage (HR: 4.70; 95% CI: 2.19–10.09; P <0.001) and PLR (HR: 2.44; 95% CI: 1.38–4.32; P =0 .002) are also independent prognostic factors for DFS of RCC patients.

**Table 4:** Univariate and multivariate analyses for variables considered for disease-free survival (DFS) (Cox proportional hazard regression model) (*n* = 541)

	DFS Univariate analysis			DFS Multivariate analysis		
Characteristics	95% CIs	HR	*P* value	95% CIs	HR	*P* value
Age (years)	1.02 to 1.05	1.03	0.002^a^	1.01 to 1.06	1.03	<0.001^b^
BMI	0.79 to 0.93	0.86	<0.001^a^	0.82 to 0.97	0.89	0.005^b^
Gender						
Male		1.00(ref.)				
Female	0.57 to 1.66	0.97	0.921^a^			
Pathological types						
Clear cell carcinoma		1.00(ref.)			1.00(ref.)	
Papillary carcinoma	1.15 to 4.80	2.35	0.019^a^	0.62 to 2.99	1.47	0.431^b^
Others	0.33 to 1.80	0.77	0.550^a^	0.32 to 1.91	0.78	0.587^b^
Fuhrman-grade						
I		1.00(ref.)				
II	0.62 to 2.68	1.28	0.504^a^			
III	1.01 to 5.86	2.44	0.047^a^			
IV	0.97 to 20.24	4.99	0.055^a^			
unknown	0.68 to 3.40	1.53	0.299^a^			
pTNM stage						
I		1.00(ref.)				
II	1.33 to 4.84	2.54	0.005^a^			
III	3.54 to 11.08	6.26	<0.001^a^			
pT status						
T1		1.00(ref.)			1.00(ref.)	
T2	1.57 to 5.03	2.81	<0.001^a^	1.29 to 4.35	2.38	0.005^b^
T3	2.20 to 8.03	4.20	<0.001^a^	0.65 to 2.72	1.33	0.437^b^
pN status						
N0		1.00(ref.)			1.00(ref.)	
N1	3.72 to 13.73	7.15	<0.001^a^	2.19 to 10.09	4.70	<0.001^b^
Urine protein						
No		1.00(ref.)				
Yes	0.44 to 3.40	1.23	0.691^a^			
Unknown	0.28 to 1.49	0.64	0.301^a^			
ALP						
Normal		1.00(ref.)				
Elevated	0.19 to 2.02	0.63	0.440^a^			
LDH						
Normal		1.00(ref.)				
Elevated	0.84 to 2.69	1.51	0.168^a^			
CRE						
Normal		1.00(ref.)				
Elevated	0.59 to 4.51	1.64	0.341^a^			
UA						
Normal		1.00(ref.)				
Elevated	0.44 to 1.69	0.86	0.654^a^			
Total protein						
Normal		1.00(ref.)				
Elevated	0.37 to 1.97	0.85	0.704^a^			
Serum globulin						
Normal		1.00(ref.)			1.00(ref.)	
Elevated	1.26 to 3.40	2.07	0.004^a^	0.69 to 2.05	1.19	0.533^b^
NLR						
< 1.85		1.00(ref.)			1.00(ref.)	
≥ 1.85	2.06 to 7.58	3.95	<0.001^a^	0.78 to 3.37	1.62	0.193^b^
PLR						
< 153		1.00(ref.)			1.00(ref.)	
≥ 153	2.54 to 6.92	4.19	<0.001^a^	1.38 to 4.32	2.44	0.002^b^
CRP/Alb						
< 0.08		1.00(ref.)			1.00(ref.)	
≥ 0.08	2.54 to 7.02	4.22	<0.001^a^	1.22 to 3.75	2.14	0.008^b^

*Abbreviation: HR* hazard ratio, *CIs* confidence intervals, *BMI* body mass index, *pTNM* pathologic tumor–node–metastasis, *ALP* alkaline phosphatase, *LDH* lactate dehydrogenase, *CRE* serum creatinine, *UA* uric acid, *LDH* lactate dehydrogenase , *NLR* neutrophil count to lymphocyte count, *PLR* platelet count to lymphocyte count, *CRP/Alb* the serum CRP level to the serum Alb level

^a^Univariate Cox proportional hazard regression

^b^Multivariate Cox proportional hazard regression

## Discussion

In this study, we retrospectively analyzed the prognostic value of CRP/Alb ratio in 570 RCC patients received radical or partial nephrectomy in our institution. Among them, 541 patients with localized (T1-3 N0/+ M0) RCC and subjected to full resection were also analyzed. The results demonstrated that CRP/Alb ratio is an independent prognostic factor for patients with RCC.

Although the basal CRP level is affected by genetic and environmental factors [20, 21], CRP is produced mainly by hepatocytes and is regulated by pro-inflammatory cytokines, especially interleukin-6 [22]. Increased CRP level has been reported in many types of cancers [23–25]. The potential mechanisms for the association of CRP with cancer have been proposed. (1) Tissue inflammation was caused by the tumor growth may result in increased CRP levels [26]. (2) The elevated CRP could be an indicative biomarker of immune responses to tumor antigens [27]. (3) Tumor cells could produce more inflammatory proteins including CRP [24] or enhanced interleukin-6 and interlukin-8 in tumor cells could indirectly increase CRP expression [28]. Jabs WJ et al. showed that activity of the IL-6/CRP network in RCC patients contributes to the acute-phase reaction in local inflammatory processes [29]. Other clinical data also showed that elevated CRP level is associated with poorer OS of RCC patients [30, 31] and CRP has a significant impact on OS of metastatic RCC patients treated with a tyrosine kinase inhibitor, either sunitinib or sorafenib [32, 33].

Hypoalbuminemia is not a perfect indicator of nutritional status because of its long half-life and the potential influence of system factors such as inflammation and stress on serum Alb. However, it is an easy, reproducible assessment and closely correlated with other markers of nutritional status [34]. In addition, serum Alb as a biomarker of protein-energy malnutrition can provide essential information that supplementary to BMI and changes in body weight, which may not accurately reflect the nutritional status due to normal limits [35]. Protein malnutrition can lead to edema, impaired organ function and immunosuppression. Moreover, preoperative hypoalbuminemia is associated with higher mortality in patients with underwent surgery for RCC [14, 36, 37].

Recently, CRP/Alb ratio has been used to predict the prognosis of several cancers [16–18]. In this study, we used the ROC analysis to yield a 0.08 cutoff value for CRP/Alb ratio for predicting OS in RCC. Compared with the other systemic inflammatory markers NLR and PLR, CRP/Alb ratio has the highest AUC value (*P* < 0.001). Univariate analyses showed that higher CRP/Alb ratio is associated with poorer prognosis (*P* < 0.001). Patients with CRP/Alb ratio ≥ 0.08 had a 5.5-higher mortality risk than patients with CRP/Alb ratio < 0.08. Multivariate analyses also showed that CRP/Alb ratio is independently predictable factor for OS of patients with RCC (*P* = 0.018). Furthermore, RCC patients at stage T1/T2, stage T3/T4, N0, N1 and M0, but M1, in the low CRP/Alb group had significantly longer OS than their counterparts in the high CRP/Alb group (*P* < 0.001, *P* < 0.001, *P* < 0.001, *P* = 0.006 and *P* < 0.001, respectively). Therefore, low CRP/Alb ratio is associated with the early stage RCC disease and high CRP/Alb ratio present the advanced or metastatic RCC suggesting that CRP/Alb ratio could be a new prognostic indicator related to the progression of RCC. More importantly, CRP/Alb ratio is also a predictor for recurrence or metastasis of localized (T1-3 N0/+ M0) RCC patients underwent full resection. In general, T1/T2 is staged as localized disease in RCC. However, patients at T1-3N1M0 stages underwent full resection may be also considered to receive a curative therapy. So far, although target reagent clinical trials for localized (T1-3N1M0) RCC patients using adjuvant therapy including axitinib are ongoing, there is no standard therapy for those patients after surgery. Therefore, looking for predict markers for full resection in localized (T1-3/N1 M0) RCC patients can avoid excessive treatment and help clinicians to identify high-risk patients for closer follow-up. Multivariate analysis of these 541 patients after adjustment for other variables including cancer stage, CRP/Alb ratio was an independent prognostic factor for DFS of patients in full resection of localized RCC (*P* = 0.008). Patients underwent full resection of localized RCC in the low CRP/Alb group showed significantly longer DFS than patients underwent full resection of localized RCC in the high CRP/Alb group (*P* < 0.001). Further analyses also suggested that patients underwent full resection at T1, T2, T3, N0 and N1 stages in the low CRP/Alb group had longer DFS than their counterparts in the high CRP/Alb group. To our best knowledge, this is the first report showing that CRP/Alb ratio can predict the DFS of localized RCC underwent full resection. These results are crucial for clinicians to make a decision for full resection of localized RCC and important to increase the accuracy of the established prognostic factors. The study also demonstrates that CRP/Alb ratio may serve as a screening method to choose the appropriate follow-up strategy for patients with localized RCC underwent full resection.

Our findings have some clinical implications. Firstly, compared with the preoperative NLR and PLR, the preoperative CRP/Alb ratio is more effective and suitable prognostic indicator in patients with RCC. Secondly, according to the preoperative CRP/Alb ratio, patients with high risk can be selected for further management and treatment. Thirdly, our result can be used to stratify patients who are more likely to respond to biomarker-based enrichment strategy in future clinical trials. Our study also has several limitations. First, it is a retrospective and single-center study, which may limit the prognostic value of the CRP/Alb ratio. Therefore, a large-scale prospective validation study is needed. Second, several other factors that are influential to inflammation such as life styles and smoking status were not included in the study. Third, DFS was recorded based on radio-examination which may be longer than the actual DFS as some patients admitted to hospital only when they had obvious symptoms. In summary, this study demonstrated that CRP/Alb ratio is an independent predictor of OS for patients with RCC and can be used to predict the relapse or metastasis of localized RCC patients underwent full resection.

## Conclusion

Overall, we demonstrate that preoperative CRP/Alb ratio is an independent prognostic marker for OS of RCC patients after radical or partial nephrectomy. In addition, preoperative CRP/Alb ratio could be used to predict DSF of localized RCC patients who underwent curative treatment and help clinicians to identify the high-risk patients for closer follow-up.

## Additional files

Additional file 1: Figure S1. The relationship of serum CRP (A), Alb (B) with OS. (TIF 274 kb)
Additional file 2: Table S1. ROC analyses for inflammation-based factors. (DOC 28 kb)
Additional file 3: Table S2. Baseline characteristics of patients with localized disease (*n* = 541). (DOC 70 kb)

## Abbreviations

ALP: Alkaline phosphatase
BMI: Body mass index
CI: Confidence interval
CRE: Serum creatinine
CRP/Alb: C-reactive protein/Albumin
DFS: Disease-free survival
GPS: Glasgow prognostic score
HR: Hazard ratio
LDH: Lactate dehydrogenase
NLR: Neutrophil count to lymphocyte count ratio
OS: Overall survival
PLR: Platelet count to lymphocyte count ratio
RCC: Renal cell cancer
UA: Uric acid

## References

[1] Siegel RL, Miller KD, Jemal A (2015). Cancer statistics, 2015. CA Cancer J Clin.

[2] Palsdottir HB, Hardarson S, Petursdottir V, Jonsson A, Jonsson E, Sigurdsson MI, Einarsson GV, Gudbjartsson T (2012). Incidental detection of renal cell carcinoma is an independent prognostic marker: results of a long-term, whole population study. J Urol.

[3] Park YH, Baik KD, Lee YJ, Ku JH, Kim HH, Kwak C (2012). Late recurrence of renal cell carcinoma >5 years after surgery: clinicopathological characteristics and prognosis. BJU Int.

[4] Ljungberg B, Bensalah K, Canfield S, Dabestani S, Hofmann F, Hora M, Kuczyk MA, Lam T, Marconi L, Merseburger AS (2015). EAU guidelines on renal cell carcinoma: 2014 update. Eur Urol.

[5] Battaglia M, Lucarelli G (2015). The role of renal surgery in the era of targeted therapy: the urologist’s perspective. Urologia.

[6] Lee-Ying R, Lester R, Heng D (2014). Current management and future perspectives of metastatic renal cell carcinoma. Int J Urol.

[7] Billia M, Volpe A, Terrone C (2011). The current TNM staging system of renal cell carcinoma: are further improvements needed?. Arch Esp Urol.

[8] Hayes DF, Allen J, Compton C, Gustavsen G, Leonard DG, McCormack R, Newcomer L, Pothier K, Ransohoff D, Schilsky RL (2013). Breaking a vicious cycle. Sci Transl Med.

[9] Tai CG, Johnson TV, Abbasi A, Herrell L, Harris WB, Kucuk O, Canter DJ, Ogan K, Pattaras JG, Nieh PT (2014). External validation of the modified Glasgow prognostic score for renal cancer. Indian J Urol.

[10] Mathias TM, Silva JF, Sapata VM, Marson FC, Zanoni JN, Silva CO (2014). Evaluation of the effects of periodontal treatment on levels of ascorbic acid in smokers. J Int Acad Periodontol.

[11] Gunduz S, Mutlu H, Tural D, Yildiz O, Uysal M, Coskun HS, Bozcuk H (2015). Platelet to lymphocyte ratio as a new prognostic for patients with metastatic renal cell cancer. Asia Pac J Clin Oncol.

[12] Yang JJ, Hu ZG, Shi WX, Deng T, He SQ, Yuan SG (2015). Prognostic significance of neutrophil to lymphocyte ratio in pancreatic cancer: a meta-analysis. World J Gastroenterol.

[13] Omae K, Kondo T, Tanabe K (2015). High preoperative C-reactive protein values predict poor survival in patients on chronic hemodialysis undergoing nephrectomy for renal cancer. Urol Oncol.

[14] Morgan TM, Tang D, Stratton KL, Barocas DA, Anderson CB, Gregg JR, Chang SS, Cookson MS, Herrell SD, Smith JJ (2011). Preoperative nutritional status is an important predictor of survival in patients undergoing surgery for renal cell carcinoma. Eur Urol.

[15] Choi Y, Park B, Jeong BC, Seo SI, Jeon SS, Choi HY, Adami HO, Lee JE, Lee HM (2013). Body mass index and survival in patients with renal cell carcinoma: a clinical-based cohort and meta-analysis. Int J Cancer.

[16] Kinoshita A, Onoda H, Imai N, Iwaku A, Oishi M, Tanaka K, Fushiya N, Koike K, Nishino H, Matsushima M (2015). The C-reactive protein/albumin ratio, a novel inflammation-based prognostic score, predicts outcomes in patients with hepatocellular carcinoma. Ann Surg Oncol.

[17] Liu X, Sun X, Liu J, Kong P, Chen S, Zhan Y, Xu D (2015). Preoperative C-reactive protein/albumin ratio predicts prognosis of patients after curative resection for gastric cancer. Transl Oncol.

[18] Zhou T, Zhan J, Hong S, Hu Z, Fang W, Qin T, Ma Y, Yang Y, He X, Zhao Y (2015). Ratio of C-reactive protein/albumin is an inflammatory prognostic score for predicting overall survival of patients with small-cell lung cancer. Sci Rep.

[19] Chen Z, Shao Y, Fan M, Zhuang Q, Wang K, Cao W, Xu X, He X (2015). Prognostic significance of preoperative C-reactive protein: albumin ratio in patients with clear cell renal cell carcinoma. Int J Clin Exp Pathol.

[20] Brull DJ, Serrano N, Zito F, Jones L, Montgomery HE, Rumley A, Sharma P, Lowe GD, World MJ, Humphries SE (2003). Human CRP gene polymorphism influences CRP levels: implications for the prediction and pathogenesis of coronary heart disease. Arterioscler Thromb Vasc Biol.

[21] Greenfield JR, Samaras K, Jenkins AB, Kelly PJ, Spector TD, Gallimore JR, Pepys MB, Campbell LV (2004). Obesity is an important determinant of baseline serum C-reactive protein concentration in monozygotic twins, independent of genetic influences. Circulation.

[22] Black S, Kushner I, Samols D (2004). C-reactive protein. J Biol Chem.

[23] Deichmann M, Benner A, Waldmann V, Bock M, Jackel A, Naher H (2000). Interleukin-6 and its surrogate C-reactive protein are useful serum markers for monitoring metastasized malignant melanoma. J Exp Clin Cancer Res.

[24] Nozoe T, Korenaga D, Futatsugi M, Saeki H, Maehara Y, Sugimachi K (2003). Immunohistochemical expression of C-reactive protein in squamous cell carcinoma of the esophagus - significance as a tumor marker. CANCER LETT.

[25] Wieland A, Kerbl R, Berghold A, Schwinger W, Mann G, Urban C (2003). C-reactive protein (CRP) as tumor marker in pediatric and adolescent patients with Hodgkin disease. Med Pediatr Oncol.

[26] Mroczko B, Groblewska M, Gryko M, Kedra B, Szmitkowski M (2010). Diagnostic usefulness of serum interleukin 6 (IL-6) and C-reactive protein (CRP) in the differentiation between pancreatic cancer and chronic pancreatitis. J Clin Lab Anal.

[27] Balkwill F, Mantovani A (2001). Inflammation and cancer: back to Virchow?. Lancet.

[28] Aggarwal BB, Gehlot P (2009). Inflammation and cancer: how friendly is the relationship for cancer patients?. Curr Opin Pharmacol.

[29] Jabs WJ, Busse M, Kruger S, Jocham D, Steinhoff J, Doehn C (2005). Expression of C-reactive protein by renal cell carcinomas and unaffected surrounding renal tissue. Kidney Int.

[30] Sim SH, Messenger MP, Gregory WM, Wind TC, Vasudev NS, Cartledge J, Thompson D, Selby PJ, Banks RE (2012). Prognostic utility of pre-operative circulating osteopontin, carbonic anhydrase IX and CRP in renal cell carcinoma. Br J Cancer.

[31] Steffens S, Kohler A, Rudolph R, Eggers H, Seidel C, Janssen M, Wegener G, Schrader M, Kuczyk MA, Schrader AJ (2012). Validation of CRP as prognostic marker for renal cell carcinoma in a large series of patients. BMC Cancer.

[32] Yasuda Y, Saito K, Yuasa T, Kitsukawa S, Urakami S, Yamamoto S, Yonese J, Takahashi S, Fukui I (2013). Prognostic impact of pretreatment C-reactive protein for patients with metastatic renal cell carcinoma treated with tyrosine kinase inhibitors. Int J Clin Oncol.

[33] Fujita T, Nishi M, Tabata K, Matsumoto K, Yoshida K, Iwamura M (2016). Overall prognostic impact of C-reactive protein level in patients with metastatic renal cell carcinoma treated with sorafenib. Anticancer Drugs.

[34] Baker JP, Detsky AS, Wesson DE, Wolman SL, Stewart S, Whitewell J, Langer B, Jeejeebhoy KN (1982). Nutritional assessment: a comparison of clinical judgement and objective measurements. N Engl J Med.

[35] Rohlman DS, Bailey SR, Anger WK, McCauley L (2001). Assessment of neurobehavioral function with computerized tests in a population of hispanic adolescents working in agriculture. Environ Res.

[36] Kim HL, Han KR, Zisman A, Figlin RA, Belldegrun AS (2004). Cachexia-like symptoms predict a worse prognosis in localized t1 renal cell carcinoma. J Urol.

[37] Kim HL, Belldegrun AS, Freitas DG, Bui MH, Han KR, Dorey FJ, Figlin RA (2003). Paraneoplastic signs and symptoms of renal cell carcinoma: implications for prognosis. J Urol.

