# Supplementary figures and images for: The C-reactive protein/albumin ratio, a validated prognostic score, predicts outcome of surgical renal cell carcinoma patients
Source: BMC Cancer. 2017 Mar 6;17:171. doi: 10.1186/s12885-017-3119-6 (PMC5339967; doi:10.1186/s12885-017-3119-6)

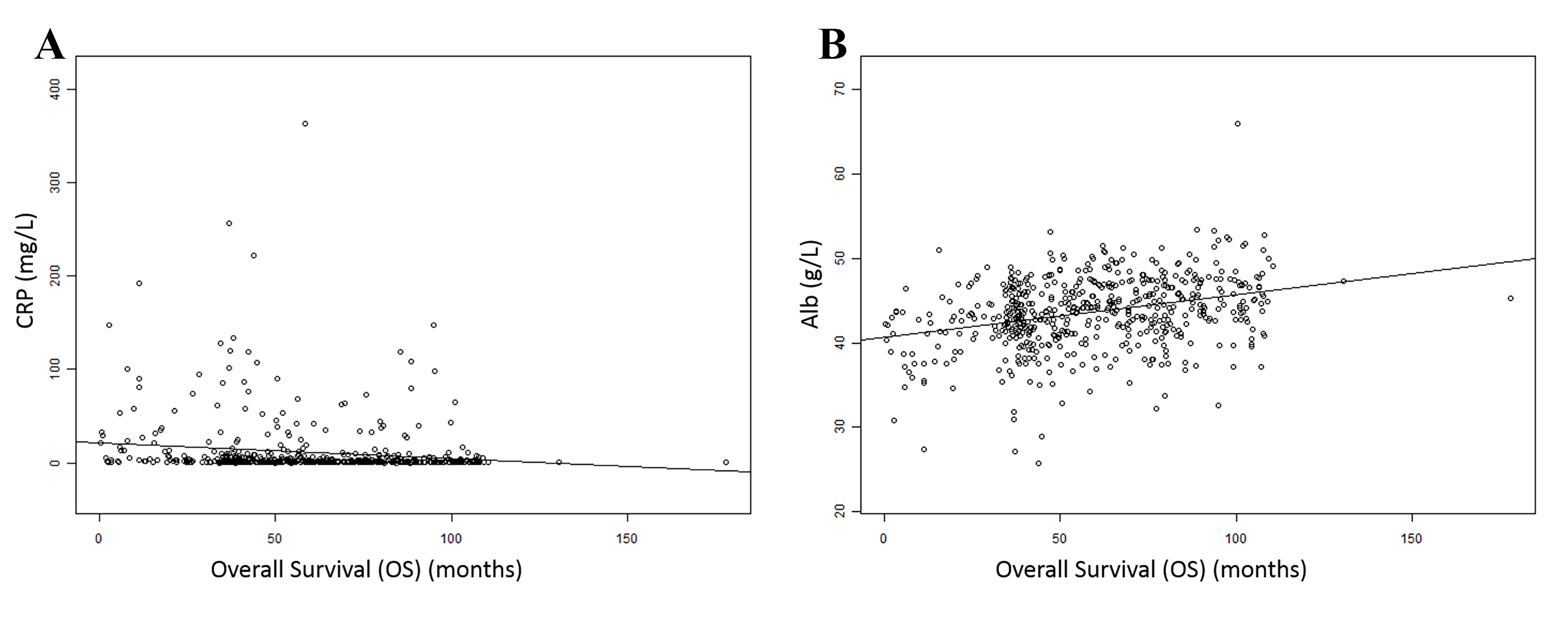

Supplement: Additional file 1: Figure S1. — The relationship of serum CRP (A), Alb (B) with OS. (TIF 274 kb) [file 12885_2017_3119_MOESM1_ESM.tif]
