# Supplementary material for: The C-reactive protein/albumin ratio, a validated prognostic score, predicts outcome of surgical renal cell carcinoma patients
Source: BMC Cancer. 2017 Mar 6;17:171. doi: 10.1186/s12885-017-3119-6 (PMC5339967; doi:10.1186/s12885-017-3119-6)
Supplement: Additional file 2: Table S1. — ROC analyses for inflammation-based factors. (DOC 28 kb) [file 12885_2017_3119_MOESM2_ESM.doc]

| variable | Cut-off value | AUC | P value |
| --- | --- | --- | --- |
| CRP/Alb | 0.08 | 0.715 | <0.001 |
| NLR | 1.85 | 0.675 | <0.001 |
| PLR | 153 | 0.704 | <0.001 |

**Additional file 2: Table S1**. ROC analyses for inflammation-based factors

Abbreviation: NLR=neutrophil count to lymphocyte count, PLR=platelet count to lymphocyte count, CRP/Alb= the serum CRP level to the serum Alb level.
