# Supplementary material for: The C-reactive protein/albumin ratio, a validated prognostic score, predicts outcome of surgical renal cell carcinoma patients
Source: BMC Cancer. 2017 Mar 6;17:171. doi: 10.1186/s12885-017-3119-6 (PMC5339967; doi:10.1186/s12885-017-3119-6)
Supplement: Additional file 3: Table S2. — Baseline characteristics of patients with localized disease (n = 541). (DOC 70 kb) [file 12885_2017_3119_MOESM3_ESM.doc]

**Additional file 3: Table S2** Baseline characteristics of patients with localized disease (n=541)

| **Characteristics** | **Cases (n=541)** | **Percentage (%)** |
| --- | --- | --- |
| **Age (years)** (Mean±SD) | 51.12±13.47 | |
| **BMI** (Mean±SD) | 23.63±3.60 | |
| **Gender** |  |  |
| Male | 363 | 67.10 |
| Female | 178 | 32.90 |
| **Pathological types** |  |  |
| clear cell carcinoma | 400 | 73.90 |
| Papillary carcinoma | 85 | 15.70 |
| others | 56 | 10.40 |
| **Fuhrman-grade** |  |  |
| I | 117 | 21.60 |
| II | 242 | 44.70 |
| III | 54 | 10.00 |
| Ⅳ | 6 | 1.10 |
| unknown | 122 | 22.60 |
| **pTNM stage** |  |  |
| I | 397 | 73.40 |
| II | 85 | 15.70 |
| III | 59 | 10.90 |
| **pT status** |  |  |
| T1 | 405 | 74.90 |
| T2 | 90 | 16.60 |
| T3 | 46 | 8.50 |
| **pN status** |  |  |
| N0 | 519 | 95.90 |
| N1 | 22 | 4.10 |
| **Urine protein** |  |  |
| No | 437 | 80.80 |
| Yes | 27 | 5.00 |
| Unknow | 77 | 14.20 |
| **ALP** |  |  |
| Normal | 502 | 92.80 |
| Elevated | 39 | 7.20 |
| **LDH** |  |  |
| Normal | 446 | 82.40 |
| Elevated | 95 | 17.60 |
| **CRE** |  |  |
| Normal | 519 | 95.90 |
| Elevated | 22 | 4.10 |
| **UA** |  |  |
| Normal | 440 | 81.30 |
| Elevated | 101 | 18.70 |
| **Total protein** |  |  |
| Normal | 482 | 89.10 |
| Elevated | 59 | 10.90 |
| **Serum globulin** |  |  |
| Normal | 350 | 64.70 |
| Elevated | 191 | 35.30 |
| **NLR** |  |  |
| ˂1.85 | 238 | 44.00 |
| ≥1.85 | 303 | 56.00 |
| **PLR** |  |  |
| ˂153 | 406 | 75.00 |
| ≥153 | 135 | 25.00 |
| **CRP/Alb** |  |  |
| ˂0.08 | 388 | 71.70 |
| ≥0.08 | 153 | 28.30 |

Abbreviation: BMI: body mass index; pTNM, pathologic tumor–node–metastasis; ALP: alkaline phosphatase; LDH: lactate dehydrogenase; CRE: serum creatinine; UA: uric acid; lactate dehydrogenase (LDH), NLR=neutrophil count to lymphocyte count, PLR=platelet count to lymphocyte count, CRP/Alb= the serum CRP level to the serum Alb level.
